# Letter to the Editor of the *Journal of Nutritional Science*

**DOI:** 10.1017/jns.2022.99

**Published:** 2023-02-21

**Authors:** Marko Kerac, Marie McGrath, James A. Berkley, Carlos S. Grijalva-Eternod, Natasha Lelijveld, Martha Mwangome, Eleanor Rogers

**Affiliations:** 1 London School of Hygiene and Tropical Medicine, Keppel Street, London WC1E 7HT, UK; 2 Emergency Nutrition Network, 69 High Street, Marlborough House, Kidlington, Oxfordshire OX5 2DN, UK; 3 Kenya Medical Research Institute (KEMRI)/Wellcome Trust Research Programme, P.O Box 230, Kilifi, Kenya; 4 UCL Institute for Global Health, 30 Guilford Street, London WC1N 1EH, UK

Dear Editor,

We are writing in response to the recently published article *Jima BR, Hassen HY, Bahwere P and Gebreyesus SH, Diagnostic ability of mid-upper arm circumference-to-length ratio in detecting wasting among infants aged 1–6 months in Ethiopia, Journal of Nutritional Science, (2022), vol. 11, e23, p1–8*.

This study addresses an important topic, focusing on small and nutritionally at-risk infants under 6 months of age (u6m) who are at high risk of wasting^(1,2)^ but for whom there are considerable, continued gaps in evidence to inform identification and management^(3)^. However, we have concerns with the study's premises, which need to be highlighted to avoid continuing replication of an unhelpful methodology. There are considerable misconceptions already in this field and this study may risk exacerbating those misunderstandings, causing further confusion. We, therefore, present some clarifications hoping readers and future researchers will find them helpful.

First, implicit in the study design is an assumption that weight-for-length (WLZ) is the gold standard for identifying wasting in infants u6m. This is incorrect. All anthropometric indicators are imperfect proxy measures of malnutrition with different strengths and weaknesses: what matters is how well they help identify infants at high risk of mortality and morbidity^(4)^. Although low WLZ indeed forms the current WHO 2013 case definition for severe malnutrition in infants u6m^(5)^, there is increasing evidence that it is a poor indicator of risk in this age group. Other indicators are likely to perform better, namely weight-for-age (WAZ) and potentially unadjusted mid-upper-arm-circumference (MUAC)^(6)^. These indicators (along with non-anthropometric criteria) are currently being examined by the WHO in a collaborative pooled analysis of multiple datasets to explore their predictive value related to functional outcomes (mortality).

A second problem is the lack of practical considerations in the study's discussion. MUAC-to-length (MUAC/L) is a complex and problematic measure considering the challenges of accurately measuring length in infants. This makes it a poor, non-viable option in health and nutrition programmes in low resource settings. Length measurement requires a lot of time, training and equipment to measure, it is difficult to do accurately as infants’ legs are naturally flexed, and it has the lowest quality data of any anthropometric measurement^(7,8)^. These factors have been influential in the development of MUAC-only programmes rather than those based on WLZ in children aged 6–59 months^(9)^.

To improve future research and evidence generation in this area, we suggest that the primary aim of future studies on anthropometric measurements for infants u6m should assess risk of mortality, morbidity or neurodevelopmental outcomes^(10,11)^. Anthropometric recovery is not an end in itself as mortality risk persists even when weight has been regained^(12)^. An alternative to ROC curves, which position WLZ as the gold standard, would be to assess anthropometric overlap using Venn diagrams^(13)^. Then to explore how different indicators or combinations of indicators predict mortality/morbidity/development outcomes. Given the age dependence of MUAC, all such analyses would benefit from stratifying the analysis into infants aged 0–6 weeks and 7 weeks – 6 months. As immunisations often occur at 6 weeks of age, this threshold is relatively easy to implement^(14,15)^.

We strongly encourage the collection and publication of data which examine anthropometry of infants u6m. However, it is essential that the considerable confusion around anthropometry is addressed so that all studies can contribute much needed evidence to the field. We hope that this short summary goes some way to bringing clarity and researchers are suitably informed and appropriately equipped to produce much needed data which will improve the outcomes for infants u6m, enabling them to not only survive but also thrive.
